# Network Pharmacology-Based Strategy for Exploring the Pharmacological Mechanism of Honeysuckle (*Lonicer japonica* Thunb.) against Newcastle Disease

**DOI:** 10.1155/2022/9265094

**Published:** 2022-04-05

**Authors:** Yi Lu, Wen A. Huang, Zhi B. He, Shan Li, Jun Liu

**Affiliations:** ^1^Medical College, Jiujiang University, Jiujiang 332005, China; ^2^School of Foreign Languages, Jiujiang University, Jiujiang 332005, China

## Abstract

**Objective:**

Newcastle disease causes huge economic losses in the global poultry industry. An efficient treatment is needed to deal with the variable immunogenicity of the Newcastle disease virus (NDV). This study utilized network pharmacology to study the potential therapeutic targets of Honeysuckle (*Lonicer japonica* Thunb.) against Newcastle disease.

**Methods:**

Venny online analysis was used to analyze the potential overlapping targets of Honeysuckle and Newcastle disease. Hub genes were obtained using the STRING database and Cytoscape 3.8.2 software. Gene Ontology (GO) functions and Kyoto Encyclopedia of Genes and Genomics (KEGG) pathway enrichment analysis using the DAVID online tool were performed on these targets.

**Results:**

Twenty-five overlapping targets were identified. The PPI network construction results included 23 nodes of 25 genes and 95 edges. It was found that the IL-6 node had the largest degree. STAT1 and IRF1, CASP9, and CASP3 had the same as well as strongest interaction strengths. GO functions, such as “cytokine activity,” had a regulatory effect on NDV. The “Toll-like receptor signaling Pathway” “Nod-like receptor signaling pathway,” “RIG-I-like receptor signaling pathway,” and “Apoptosis,” which were obtained using KEGG analysis, also indicated that these pathways can act on NDV to enhance immune function.

**Conclusions:**

In this study, the potential targets and mechanisms of action of Honeysuckle against Newcastle disease were explored through network pharmacology, which provided a theoretical basis for the treatment of Newcastle disease and provided new ideas for the development of traditional Chinese medicine for the poultry industry.

## 1. Introduction

Newcastle disease is one of the most dangerous diseases affecting the poultry industry worldwide. It is caused by the Newcastle disease virus (NDV), which can be rapidly transmitted through contact [1, 2]. It can cause heart failure [3] and severe inflammation [4]. Injection of inactivated vaccine is the main way to prevent and control Newcastle disease (ND) [5]. After vaccination, large-scale epidemics of Newcastle disease were controlled in many areas; however, due to different age groups, immune levels, and the variability of NDV immunogens, small-scale epidemics of Newcastle disease still occur frequently in the flock [6, 7]. To reduce the frequency of small outbreaks of Newcastle disease and the consequent economic losses, a safe and effective treatment method is needed.

Traditional Chinese medicine (TCM) has played an important role in preventing and treating diseases in China for more than 2,000 years [8]. Recently, with the emergence of coronavirus disease 2019 (COVID-19), TCM has attracted more and more attention from international medical researchers [9, 10]. For example, furanocoumarins activate multiple signaling pathways, leading to apoptosis, autophagy, antioxidant, antimetastasis, and cell cycle arrest in malignant cells [11]. Martínez et al. considered the hemp plant as a possible source of new functional food ingredients and nutraceuticals that might eventually be useful to treat or even prevent gastrointestinal conditions [12]. Honeysuckle (*Lonicer japonica* Thunb.), a TCM, is mainly distributed in the Guangdong, Shandong, Henan, Hebei, and Hainan provinces, as well as Himalayas in China. It is rich in organic acids, flavonoids, volatile oils, and iridoid glycosides and has antiviral and anti-inflammatory effects [13–15]. However, research on the pharmacological effects of Honeysuckle is not perfect. There are few basic clinical studies on Honeysuckle against Newcastle disease [16].

Network pharmacology is based on the analysis of the “drug-target-gene-disease” interaction network, which can reveal the effect mechanism of the synergistic effect of multimolecular drugs on the human body. The development of network pharmacology technology has provided great convenience for the study of the multicomponent, multipoint, and multichannel effect mechanism of Chinese medicine and Chinese medicine compounds in the treatment of diseases. Based on the technology of network pharmacology, this study systematically analyzes the potential therapeutic targets and molecular mechanisms of Honeysuckle in the treatment of Newcastle disease and provides a theoretical basis for the clinical application of Honeysuckle.

## 2. Materials and Methods

### 2.1. Identification of the Active Ingredients of Honeysuckle

The Chinese pinyin name “Jinyinhua” was the key word input in the “Herb Name” column in the Traditional Chinese Medicine Systems Pharmacology (TCMSP) database (https://tcmspw.com/tcmsp.php). Thereafter, relevant parameters such as oral bioavailability (OB) ≥ 30% and drug-likeness (DL) ≥ 0.18 were set for the active ingredients of Honeysuckle.

### 2.2. Acquisition of Honeysuckle Gene Names

To obtain the target names of Honeysuckle, the Mol ID of the active ingredients in Honeysuckle was entered into the target information as a screening condition. The target names were input in the UniProt (https://www.uniprot.org/) database to obtain the gene names of Honeysuckle. The “Reviewed” option was selected as a constraint on the method used in the UniProt database.

### 2.3. Obtaining Gene Names of Newcastle Disease

The gene symbols of Newcastle disease were obtained by entering the keyword “Newcastle disease” into the GeneCards (https://www.genecards.org) database for retrieval. Thereafter, these gene symbols were imported into the UniProt database to obtain the gene names of Newcastle disease. The filter criteria were the same as those used for the UniProt database in Section 2.2.

### 2.4. Getting the Overlapping Targets of Honeysuckle against Newcastle Disease

From the gene names of Honeysuckle and ND, the overlapping gene names were obtained using Venny online analysis (https://bioinfogp.cnb.csic.es/tools/venny/index.html) to identify potential targets for therapy.

### 2.5. Honeysuckle—The Active Ingredients of Honeysuckle-Targets-Newcastle Disease Network Construction

Honeysuckle, the active ingredients of Honeysuckle, the overlapping targets, and Newcastle Disease, is both the source and target node. The two nodes are imported into Cytoscape 3.8.2 to analyze the relationship. Moreover, these were grouped into 1, 2, 3, and 4 attributes. Property 1 was set to red, triangle; property 2 to white, hexagon; property 3 to orange, square; and property 4 to pink, diamond.

### 2.6. PPI Network Construction and HUB Gene Retrieval

First, we selected the “Multiple Proteins” option in the left column and then entered the overlapping targets obtained in 2.4 in the “List of Names” box in the right column, completed in the STRING database (https://www.string-db.org/) home page “Search.” The species constraint was “*Gallus gallus*.” In addition, we set the confidence of protein interaction >0.4 for predicting protein-protein interactions. Last, we inserted the resulting PPI network into Cytoscape 3.8.2 using MCODE analysis and cytoHubba plug-ins to verify the top 10 hub genes.

### 2.7. GO Functional and KEGG Pathway Enrichment Analyses

The DAVID 6.8 (https://david.ncifcrf.gov) online tool was used to perform the Gene Ontology (GO) function and Kyoto Encyclopedia of Genes and Genomes (KEGG) pathway enrichment analysis. Thereafter, “Official Gene Symbol” was selected and “chicken” was set as the species selection. The GO terms were divided into three categories, namely, the biological process (BP), molecular functional (MF), and cellular component (CC), and were displayed using a bar chart. *P* value <0.05 and FDR <0.05 were used as the screening standards.

## 3. Results

### 3.1. Overlapping Targets of Honeysuckle against Newcastle Disease

**Figure 1:**
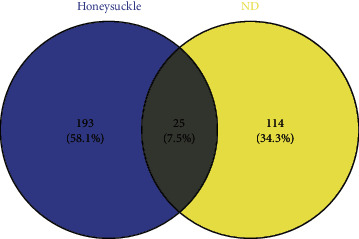
The overlapping genes in Venny diagram.

**Figure 2:**
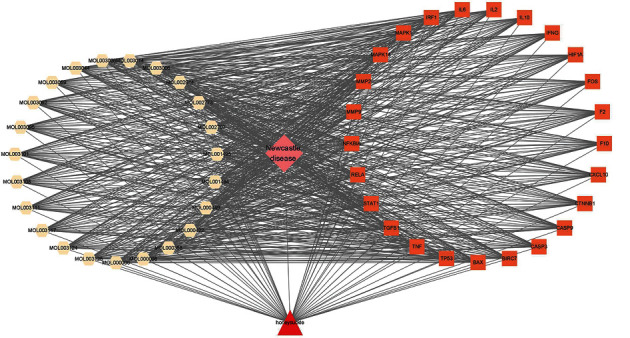
Honeysuckle—the active ingredients of the Honeysuckle-Targets-Newcastle disease network. The red shape triangle represents Honeysuckle, the white shape hexagon represents the active ingredients of Honeysuckle, the orange shape square represents the targets, and the pink shape diamond represents Newcastle disease. The line between two nodes represents the interaction.

A total of 23 active ingredients and 218 corresponding targets of Honeysuckle were retrieved from the TCMSP database (Table 1, Figure 1). One hundred and forty-two related gene symbols were obtained through searching “Newcastle disease virus” in the GeneCards database (Table S1). By converting these gene symbols, 139 gene names were obtained. To identify the potential targets of Honeysuckle against Newcastle disease, 25 overlapping targets were obtained using Venny online analysis (Figure 1, Table 2). The “Honeysuckle-Targets-Newcastle disease” network was obtained (Figure 2).

### 3.2. Protein-Protein Interaction (PPI) Network Analysis

**Figure 3:**
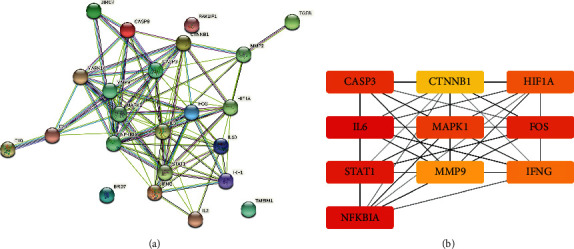
The protein-protein interaction network of overlapping targets. (a) Result from the STRING online tool. Different colors of lines indicate a different source of evidence: light blue, curated databases; rose, experimentally determined; green, gene neighborhood; red, gene fusions; dark blue, gene co-occurrence; light green, text mining; black, coexpression; purple, protein homology. (b) Top 10 hub genes of the potential targets of Honeysuckle against Newcastle disease. The darker the color, the greater the degree.

The overlapping targets were analyzed in the STRING database. The analysis results included 23 nodes and 95 edges in the network (among them, no proteins by the names BAX, TNF, and CXCL10 were detected in *Gallus gallus*) (Figure 3(a), Table S2). By further visualization and analysis of the PPI network with Cytoscape 3.8.2, it was found that there were 11 nodes whose degree was greater than or equal to 9.5 (average score) (Table S2). Among them, IL-6 had the largest degree (degree = 16), whereas the F10 node and TGFBI node had the smallest degrees (degree = 1). According to the combined score in the edge table, it was found that there were 77 edges whose combined score was greater than or equal to 0.68 (average score) (Table S3). Among them, STAT1 and IRF1, CASP9, and CASP3 had the same as well as strongest interaction strengths (score = 0.989). The interaction between MAPK1 and IL-10 had the smallest combined score (0.408). After MCODE and cytoHubba plug-in analysis in Cytoscape 3.8.2, the top 10 hub genes were obtained. The results included HIF1A, CTNNB1, IL-6, FOS, CASP3, MMP9, MAPK1, IFNG, NFKBIA, and STAT1 (Figure 3(b)).

### 3.3. GO Functional and KEGG Pathway Enrichment Analysis of Honeysuckle

**Figure 4:**
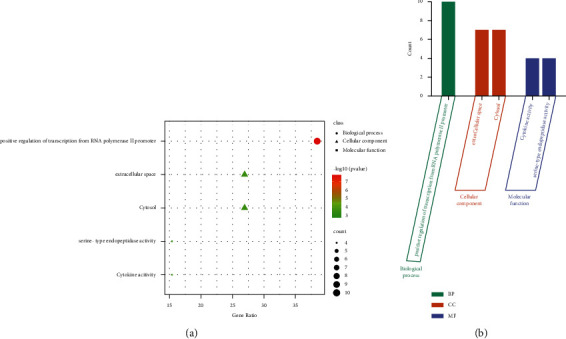
GO analysis of potential targets of Honeysuckle against Newcastle disease. (a) Enrichment dot bubble diagram. (b) Enrichment histogram. GO, gene ontology; BP, biological processes; CC, cellular components; MF, molecular functions.

**Figure 5:**
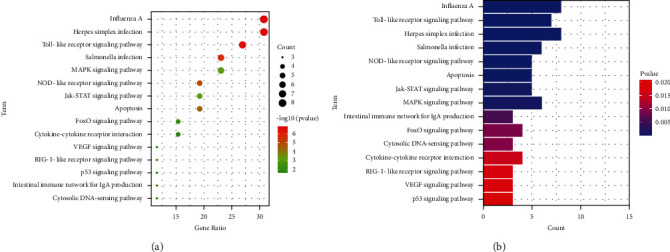
KEGG analysis of potential targets of Honeysuckle against Newcastle disease. (a) Enrichment dot bubble diagram. (b) Enrichment histogram. KEGG, Kyoto Encyclopedia of Genes and Genomes.

Using the DAVID online tool, GO functional enrichment (Figure 4) and KEGG pathway analyses (Figure 5) were conducted. It was found that the overlapping targets were enriched in five GO terms (Figure 4, Table S4). Among the GO enrichment analyses, the *P* value of “positive regulation of transcription from RNA polymerase II promoter” was the smallest (Figure 4(a)). GO terms mainly involved biological processes, cell components, and molecular functions. Of these five GO terms, one belongs to the biological process (“positive regulation of transcription from RNA polymerase II promoter.”), two belong to cellular component (“extracellular space” and “cytosol”), and two belong to molecular function (“cytokine activity” and “serine-type endopeptidase activity”) (Figure 4(b)).

Fifteen KEGG signaling pathways (*P* < 0.01) were screened according to the overlapping genes (Figure 5, Table S5). In the KEGG pathway enrichment analysis, the *P* value of the “Influenza A” pathway was the smallest compared to that of other pathways (Figure 5(a)). “Influenza A” and “Herpes simplex infection” pathways had the highest gene counts; “VEGF pathway,” “p53 signaling,” “Intestinal immune network for IgA production,” “Cytosolic DNA-sensing pathway,” and “RIG-I-like receptor signaling pathway” had the lowest gene counts in KEGG analysis, which was performed on the enriched bar graph (Figure 5(b)).

## 4. Discussion

Given the effect of clearing heat and detoxification [13, 15], the antiviral effects of Honeysuckle have been reported in the literature. One of the active ingredients of Honeysuckle was used to treat COVID-19 [17]. As one of the ingredients in the prescription, Honeysuckle has a specific role in preventing or treating Newcastle disease [16]; however, the mechanism of Honeysuckle against Newcastle disease has not been reported. In this study, a network pharmacology-based biological system network analysis was conducted to explore the potential targets and key signaling pathways of Honeysuckle against Newcastle disease, providing a new direction for the treatment of Newcastle disease.

Our results show that there are 23 active ingredients in Honeysuckle, six of which incorporate beta-carotene [18], chryseriol [19], beta-sitosterol [20], kaempferol [21], stigmasterol [22], luteolin [23], and quercetin [24], which are useful in inhibiting inflammation. Li et al. proved that luteolin protects the heart by participating in oxidative stress response and inflammation [25]. Using Venny online analysis, 25 overlapping targets were obtained, and the relevant literature showed that Honeysuckle could regulate certain potential targets. Researchers found that a water-soluble polysaccharide from Honeysuckle could increase the expression of anti-inflammatory factor IL-10 and enhance immune function, proving that Honeysuckle can regulate IL-10 [26]. In addition, Su et al. found that the ethanol extract of Honeysuckle alleviated diarrhea in mice, alleviated damage to multiple organs, and significantly inhibited the expression of proinflammatory factors such as IL-6. It indicated that Honeysuckle has good anti-inflammatory properties [14]. An experimental study showed that adding Honeysuckle extract could reduce the concentration of IL-2 in the middle stage of heat stress; it indicated that Honeysuckle extract had effects on the antioxidant status and immune function of cows [27]. Furthermore, there is evidence that *β*-carotene can reduce skin inflammation by inhibiting the expression of inflammatory factors and reducing the activity of MMP9 in the ox-AD mouse model [28]. Luteolin could inhibit lipid-induced liver inflammation by reducing the expression of IL-10 and IL-6 and play a protective role in the male C57BL/6 mice liver and primary mouse hepatocytes [29]. To investigate the effect of autophagy and apoptosis on NDV replication after NDV infection in chicken tissues and cells, researchers found that decreasing cleavage of caspase 3 can enhance autophagy and promote cell survival and NDV replication [30].

Twenty-three overlapping targets and 10 hub targets were obtained using the STRING database and Cytoscape 3.8.2. These targets have certain relationships with each other. The literature proves that potential targets can interact with each other. To explore the possible mechanism of the rheumatoid arthritis- (RA-) related abnormal glucose metabolism, researchers showed that the apoptosis-related enzyme Caspase-3 was significantly increased with upregulation of IL-6 expression [31]. MMP9 released after nerve injury is involved in the activation of microglia, leading to the release of IL-6 by microglia [32].

Through GO and KEGG analyses of 25 targets, these potential targets were allocated to different enrichment functions and pathways. There is an induced signal cascade that promotes apoptosis and cytokine secretion, and these signals cascade to promote NDV proliferation [33]. It indicates that “cytokine activity” can be implicated in resistance to NDV. According to KEGG pathway analysis, the results included the “Toll-like receptor signaling pathway,” “Nod-like receptor signaling pathway,” “RIG-I-like receptor signaling pathway,” “Apoptosis,” and other signaling pathways. The pattern recognition receptors (PRRs) involved in pathogen recognition in the host include Toll-like receptors (TLRs), RIG-I-like receptors, and Nod-like receptors [34]. The innate immune system recognizes pathogen-associated molecular patterns (PAMPs) through PRRs, which play a key role in pathogen recognition and the initiation of protective immune responses [35]. The TLR signaling pathway is an innate immune defense, and IRF7 is a key gene in this pathway. Studies have shown that the expression levels of ISTAT1, IFN-*α*, and IFN-*β* increased after IRF7 is induced by stimulation of CEF cells, suggesting that IRF7 may be involved in the TLR signaling pathway [36].

## 5. Conclusions

Based on network pharmacology, this study explored the pharmacokinetic mechanism of Honeysuckle against Newcastle disease and provided a theoretical basis for the preliminary test of Honeysuckle against Newcastle disease by retrieving various effective components of TCM, acquiring multiple targets, and exploring multiple pathways.

## Figures and Tables

**Table 1:** Information of *Corydalis decumbens* (Thunb.) Pers. ingredients.

Molecular ID	Molecular name	OB (%)	DL
MOL001494	Mandenol	42.00	0.19
MOL001495	Ethyl linolenate	46.10	0.20
MOL002707	Phytofluene	43.18	0.50
MOL002914	Eriodyctiol (flavanone)	41.35	0.24
MOL003006	(-)-(3R,8S,9R,9aS,10aS)-9-Ethenyl-8-(beta-D-glucopyranosyloxy)-2,3,9,9a,10,10a-hexahydro-5-oxo-5H,8H-pyrano[4,3-d]oxazolo[3,2-a]pyridine-3-carboxylic acid_qt	87.47	0.23
MOL003014	Secologanic dibutylacetal_qt	53.65	0.29
MOL002773	Beta-carotene	37.18	0.58
MOL003036	ZINC03978781	43.83	0.76
MOL003044	Chryseriol	35.85	0.27
MOL003059	Kryptoxanthin	47.25	0.57
MOL003062	4,5′-Retro-beta.,.beta.-carotene-3,3′-dione, 4′,5′-didehydro-	31.22	0.55
MOL003095	5-Hydroxy-7-methoxy-2-(3,4,5-trimethoxyphenyl)chromone	51.96	0.41
MOL003101	7-Epi-vogeloside	46.13	0.58
MOL003108	Caeruloside C	55.64	0.73
MOL003111	Centauroside_qt	55.79	0.50
MOL003117	Ioniceracetalides B_qt	61.19	0.19
MOL003124	Xylostosidine	43.17	0.64
MOL003128	Dinethylsecologanoside	48.46	0.48
MOL000358	Beta-sitosterol	36.91	0.75
MOL000422	Kaempferol	41.88	0.24
MOL000449	Stigmasterol	43.83	0.76
MOL000006	Luteolin	36.16	0.25
MOL000098	Quercetin	46.43	0.28

**Table 2:** Overlapping targets in Venny analysis.

No.	Gene name
1	F2
2	CASP9
3	MMP2
4	CASP3
5	CTNNB1
6	MAPK14
7	F10
8	BAX
9	TGFB1
10	RELA
11	TNF
12	STAT1
13	MMP9
14	MAPK1
15	IL-10
16	IL-6
17	TP53
18	NFKBIA
19	IL-2
20	IFNG
21	BIRC7
22	FOS
23	HIF1A
24	CXCL10
25	IRF1

## Data Availability

The data used to support the findings of this study are included within the Supplementary Materials.

## Supplementary Materials

Supplementary Materials Table S1. Information of NDV gene from the GeneCards database. Table S2. Degree information of the protein-protein interaction (PPI) network. Table S3. Combined score information of the protein-protein interaction (PPI) network. Table S4. GO terms information. Table S5. KEGG terms information.
