# Supplementary material for: Network Pharmacology-Based Strategy for Exploring the Pharmacological Mechanism of Honeysuckle (Lonicer japonica Thunb.) against Newcastle Disease
Source: Evid Based Complement Alternat Med. 2022 Apr 5;2022:9265094. doi: 10.1155/2022/9265094 (PMC9005276; doi:10.1155/2022/9265094)
Supplement: Supplementary Materials — Table S1. Information of NDV gene from the GeneCards database. Table S2. Degree information of the protein-protein interaction (PPI) network. Table S3. Combined score information of the protein-protein interaction (PPI) network. Table S4. GO terms information. Table S5. KEGG terms information. [file 9265094.f1.pdf]

Table S1

Gene Symbol	Description
YJU2B	YJU2 Splicing Factor Homolog B
IFNA1	Interferon Alpha 1
IFNB1	Interferon Beta 1
IRF7	Interferon Regulatory Factor 7
DDX58	DExD/H-Box Helicase 58
NEU1	Neuraminidase 1
IRF3	Interferon Regulatory Factor 3
MAVS	Mitochondrial Antiviral Signaling Protein
EIF2AK2	Eukaryotic Translation Initiation Factor 2 Alpha Kinase 2
EIF2S1	Eukaryotic Translation Initiation Factor 2 Subunit Alpha
IVNS1ABP	Influenza Virus NS1A Binding Protein
TLR3	Toll Like Receptor 3
TNF	Tumor Necrosis Factor
TP53	Tumor Protein P53
NFKBIA	NFKB Inhibitor Alpha
CASP3	Caspase 3
CD36	CD36 Molecule
CASP9	Caspase 9
TNFSF10	TNF Superfamily Member 10
NCR2	Natural Cytotoxicity Triggering Receptor 2
IFI27	Interferon Alpha Inducible Protein 27
NCR1	Natural Cytotoxicity Triggering Receptor 1
CXCL10	C-X-C Motif Chemokine Ligand 10
BAX	BCL2 Associated X, Apoptosis Regulator
CARD11	Caspase Recruitment Domain Family Member 11
CTNNB1	Catenin Beta 1
MMP7	Matrix Metallopeptidase 7
RAN	RAN, Member RAS Oncogene Family
KPNB1	Karyopherin Subunit Beta 1
UMOD	Uromodulin
IRF1	Interferon Regulatory Factor 1
ATM	ATM Serine/Threonine Kinase
MMP9	Matrix Metallopeptidase 9
MMP2	Matrix Metallopeptidase 2
MAPK1	Mitogen-Activated Protein Kinase 1
MAPK14	Mitogen-Activated Protein Kinase 14
NFKB1	Nuclear Factor Kappa B Subunit 1
TGFB1	Transforming Growth Factor Beta 1
RELA	RELA Proto-Oncogene, NF-KB Subunit
RAC1	Rac Family Small GTPase 1
HIF1A	Hypoxia Inducible Factor 1 Subunit Alpha
KLK3	Kallikrein Related Peptidase 3

VHL	Von Hippel-Lindau Tumor Suppressor
TIMP1	TIMP Metallopeptidase Inhibitor 1
BSG	Basigin (Ok Blood Group)
RNF5	Ring Finger Protein 5
PPA1	Inorganic Pyrophosphatase 1
RECK	Reversion Inducing Cysteine Rich Protein With Kazal Motifs
BIRC7	Baculoviral IAP Repeat Containing 7
CTRL	Chymotrypsin Like
IRF5	Interferon Regulatory Factor 5
TBK1	TANK Binding Kinase 1
STAT1	Signal Transducer And Activator Of Transcription 1
IFIH1	Interferon Induced With Helicase C Domain 1
SEC14L1	SEC14 Like Lipid Binding 1
ATR	ATR Serine/Threonine Kinase
IFNG	Interferon Gamma
IFN1@	Interferon, Type 1, Cluster
TRIM63	Tripartite Motif Containing 63
IKBKE	Inhibitor Of Nuclear Factor Kappa B Kinase Subunit Epsilon
IRF2	Interferon Regulatory Factor 2
TLR7	Toll Like Receptor 7
PITX1	Paired Like Homeodomain 1
IL12A	Interleukin 12A
MX1	MX Dynamin Like GTPase 1
PPP1CA	Protein Phosphatase 1 Catalytic Subunit Alpha
UBE2I	Ubiquitin Conjugating Enzyme E2 I
PPP1CB	Protein Phosphatase 1 Catalytic Subunit Beta
TICAM1	Toll Like Receptor Adaptor Molecule 1
PIAS1	Protein Inhibitor Of Activated STAT 1
HES1	Hes Family BHLH Transcription Factor 1
FURIN	Furin, Paired Basic Amino Acid Cleaving Enzyme
IL10	Interleukin 10
DDX3X	DEAD-Box Helicase 3 X-Linked
TNFAIP3	TNF Alpha Induced Protein 3
FKBP5	FKBP Prolyl Isomerase 5
IRF9	Interferon Regulatory Factor 9
TLR9	Toll Like Receptor 9
HPGDS	Hematopoietic Prostaglandin D Synthase
PCSK6	Proprotein Convertase Subtilisin/Kexin Type 6
PCSK5	Proprotein Convertase Subtilisin/Kexin Type 5
MGAT5	Alpha-1,6-Mannosylglycoprotein 6-Beta-N-Acetylglucosaminyltransferase
CERS2	Ceramide Synthase 2
ILF3	Interleukin Enhancer Binding Factor 3
CUEDC2	CUE Domain Containing 2
TFPT	TCF3 Fusion Partner

MIR324	MicroRNA 324
CREBBP	CREB Binding Protein
EP300	E1A Binding Protein P300
ITCH	Itchy E3 Ubiquitin Protein Ligase
UBQLN1	Ubiquilin 1
PCBP2	Poly(RC) Binding Protein 2
RNF135	Ring Finger Protein 135
IFIT2	Interferon Induced Protein With Tetratricopeptide Repeats 2
ZC3HAV1	Zinc Finger CCCH-Type Containing, Antiviral 1
FOS	Fos Proto-Oncogene, AP-1 Transcription Factor Subunit
TLR4	Toll Like Receptor 4
F10	Coagulation Factor X
F2	Coagulation Factor II, Thrombin
USP15	Ubiquitin Specific Peptidase 15
STAT2	Signal Transducer And Activator Of Transcription 2
EEF1A2	Eukaryotic Translation Elongation Factor 1 Alpha 2
ACLY	ATP Citrate Lyase
UBA1	Ubiquitin Like Modifier Activating Enzyme 1
RPA1	Replication Protein A1
KIF5B	Kinesin Family Member 5B
PVR	PVR Cell Adhesion Molecule
HSPA1A	Heat Shock Protein Family A (Hsp70) Member 1A
HSPA1L	Heat Shock Protein Family A (Hsp70) Member 1 Like
TRIM25	Tripartite Motif Containing 25
RPS27A	Ribosomal Protein S27a
HSPA1B	Heat Shock Protein Family A (Hsp70) Member 1B
TRIM22	Tripartite Motif Containing 22
NEU3	Neuraminidase 3
ST3GAL4	ST3 Beta-Galactoside Alpha-2,3-Sialyltransferase 4
MX2	MX Dynamin Like GTPase 2
LBR	Lamin B Receptor
IL2	Interleukin 2
PUM1	Pumilio RNA Binding Family Member 1
IFNA2	Interferon Alpha 2
PUM2	Pumilio RNA Binding Family Member 2
IL2RA	Interleukin 2 Receptor Subunit Alpha
IL6	Interleukin 6
NOD2	Nucleotide Binding Oligomerization Domain Containing 2
CD274	CD274 Molecule
SLC7A5	Solute Carrier Family 7 Member 5
LGALS1	Galectin 1
CCL5	C-C Motif Chemokine Ligand 5
ATG16L1	Autophagy Related 16 Like 1
PRL	Prolactin

SFRP1	Secreted Frizzled Related Protein 1
SLC3A2	Solute Carrier Family 3 Member 2
GLYAT	Glycine-N-Acyltransferase
IFRD1	Interferon Related Developmental Regulator 1
IL11	Interleukin 11
CSF3	Colony Stimulating Factor 3
LGR4	Leucine Rich Repeat Containing G Protein-Coupled Receptor 4
DHX58	DExH-Box Helicase 58
ANP32B	Acidic Nuclear Phosphoprotein 32 Family Member B
CLEC4A	C-Type Lectin Domain Family 4 Member A
ABO	ABO, Alpha 1-3-N-Acetylgalactosaminyltransferase And Alpha 1-3-Galactosyltransferase
MIR485	MicroRNA 485

2 **Table S1 Information of NDV gene from GeneCards Database**

Table S2

No.	Target name	Degree
1	F10	1
2	TGFBI	1
3	BIRC7	3
4	IL2	4
5	F2	6
6	MMP2	7
7	IRF1	7
8	CASP9	8
9	MAPK14	9
10	IL10	12
11	MMP9	12
12	IFNG	12
13	HIF1A	12
14	CTNNB1	12
15	FOS	12
16	STAT1	13
17	NFKBIA	14
18	MAPK1	14
19	CASP3	15
20	IL6	16

Table S2 Degree information of protein-protein interaction (PPI) network

Table S3

No.	shared name	combined_score
1	BIRC7 (interacts with) CASP9	0.827
2	BIRC7 (interacts with) NFKBIA	0.482
3	BIRC7 (interacts with) CASP3	0.735
4	CASP3 (interacts with) MAPK14	0.764
5	CASP3 (interacts with) IL10	0.658
6	CASP3 (interacts with) CASP9	0.989
7	CASP3 (interacts with) MAPK1	0.804
8	CASP3 (interacts with) MMP2	0.568
9	CASP3 (interacts with) BIRC7	0.735
10	CASP3 (interacts with) MMP9	0.759
11	CASP3 (interacts with) STAT1	0.594
12	CASP3 (interacts with) F2	0.444
13	CASP3 (interacts with) IFNG	0.617
14	CASP3 (interacts with) IL6	0.843
15	CASP3 (interacts with) HIF1A	0.625
16	CASP3 (interacts with) CTNNB1	0.983
17	CASP3 (interacts with) FOS	0.531
18	CASP3 (interacts with) NFKBIA	0.742
19	CASP9 (interacts with) MAPK14	0.494
20	CASP9 (interacts with) MMP9	0.553
21	CASP9 (interacts with) CTNNB1	0.554
22	CASP9 (interacts with) IL6	0.563
23	CASP9 (interacts with) NFKBIA	0.566
24	CASP9 (interacts with) MAPK1	0.613
25	CASP9 (interacts with) BIRC7	0.827
26	CASP9 (interacts with) CASP3	0.989
27	CTNNB1 (interacts with) MAPK14	0.753
28	CTNNB1 (interacts with) CASP9	0.554
29	CTNNB1 (interacts with) MAPK1	0.649
30	CTNNB1 (interacts with) MMP2	0.633
31	CTNNB1 (interacts with) MMP9	0.791
32	CTNNB1 (interacts with) STAT1	0.442
33	CTNNB1 (interacts with) IFNG	0.431
34	CTNNB1 (interacts with) IL6	0.625
35	CTNNB1 (interacts with) HIF1A	0.91
36	CTNNB1 (interacts with) NFKBIA	0.495
37	CTNNB1 (interacts with) FOS	0.885
38	CTNNB1 (interacts with) CASP3	0.983
39	F10 (interacts with) F2	0.939
40	F2 (interacts with) MAPK14	0.495
41	F2 (interacts with) MAPK1	0.701
42	F2 (interacts with) MMP9	0.662

43	F2 (interacts with) CASP3	0.444
44	F2 (interacts with) IL6	0.571
45	F2 (interacts with) F10	0.939
46	FOS (interacts with) MAPK14	0.779
47	FOS (interacts with) IL10	0.48
48	FOS (interacts with) IRF1	0.547
49	FOS (interacts with) MAPK1	0.877
50	FOS (interacts with) MMP9	0.574
51	FOS (interacts with) STAT1	0.863
52	FOS (interacts with) IFNG	0.598
53	FOS (interacts with) IL6	0.88
54	FOS (interacts with) HIF1A	0.543
55	FOS (interacts with) CTNNB1	0.885
56	FOS (interacts with) CASP3	0.531
57	FOS (interacts with) NFKBIA	0.631
58	HIF1A (interacts with) IL10	0.479
59	HIF1A (interacts with) IRF1	0.578
60	HIF1A (interacts with) MAPK1	0.595
61	HIF1A (interacts with) MMP2	0.658
62	HIF1A (interacts with) MMP9	0.655
63	HIF1A (interacts with) STAT1	0.447
64	HIF1A (interacts with) IFNG	0.534
65	HIF1A (interacts with) IL6	0.652
66	HIF1A (interacts with) NFKBIA	0.473
67	HIF1A (interacts with) FOS	0.543
68	HIF1A (interacts with) CASP3	0.625
69	HIF1A (interacts with) CTNNB1	0.91
70	IFNG (interacts with) IL10	0.97
71	IFNG (interacts with) IRF1	0.866
72	IFNG (interacts with) MAPK1	0.412
73	IFNG (interacts with) MMP9	0.63
74	IFNG (interacts with) STAT1	0.943
75	IFNG (interacts with) CTNNB1	0.431
76	IFNG (interacts with) HIF1A	0.534
77	IFNG (interacts with) NFKBIA	0.593
78	IFNG (interacts with) FOS	0.598
79	IFNG (interacts with) CASP3	0.617
80	IFNG (interacts with) IL2	0.868
81	IFNG (interacts with) IL6	0.967
82	IL10 (interacts with) MAPK1	0.408
83	IL10 (interacts with) MMP2	0.475
84	IL10 (interacts with) HIF1A	0.479
85	IL10 (interacts with) FOS	0.48
86	IL10 (interacts with) IRF1	0.619

87	IL10 (interacts with) CASP3	0.658
88	IL10 (interacts with) NFKBIA	0.669
89	IL10 (interacts with) MMP9	0.743
90	IL10 (interacts with) STAT1	0.823
91	IL10 (interacts with) IL2	0.957
92	IL10 (interacts with) IFNG	0.97
93	IL10 (interacts with) IL6	0.973
94	IL2 (interacts with) IL10	0.957
95	IL2 (interacts with) STAT1	0.564
96	IL2 (interacts with) IFNG	0.868
97	IL2 (interacts with) IL6	0.952
98	IL6 (interacts with) MAPK14	0.53
99	IL6 (interacts with) IL10	0.973
100	IL6 (interacts with) CASP9	0.563
101	IL6 (interacts with) IRF1	0.591
102	IL6 (interacts with) MAPK1	0.552
103	IL6 (interacts with) MMP2	0.627
104	IL6 (interacts with) MMP9	0.851
105	IL6 (interacts with) STAT1	0.941
106	IL6 (interacts with) F2	0.571
107	IL6 (interacts with) IFNG	0.967
108	IL6 (interacts with) CTNNB1	0.625
109	IL6 (interacts with) HIF1A	0.652
110	IL6 (interacts with) CASP3	0.843
111	IL6 (interacts with) NFKBIA	0.85
112	IL6 (interacts with) FOS	0.88
113	IL6 (interacts with) IL2	0.952
114	IRF1 (interacts with) IL10	0.619
115	IRF1 (interacts with) NFKBIA	0.545
116	IRF1 (interacts with) FOS	0.547
117	IRF1 (interacts with) HIF1A	0.578
118	IRF1 (interacts with) IL6	0.591
119	IRF1 (interacts with) IFNG	0.866
120	IRF1 (interacts with) STAT1	0.989
121	MAPK1 (interacts with) MAPK14	0.879
122	MAPK1 (interacts with) IL10	0.408
123	MAPK1 (interacts with) CASP9	0.613
124	MAPK1 (interacts with) IFNG	0.412
125	MAPK1 (interacts with) MMP2	0.437
126	MAPK1 (interacts with) IL6	0.552
127	MAPK1 (interacts with) MMP9	0.561
128	MAPK1 (interacts with) HIF1A	0.595
129	MAPK1 (interacts with) CTNNB1	0.649
130	MAPK1 (interacts with) F2	0.701

131	MAPK1 (interacts with) NFKBIA	0.778
132	MAPK1 (interacts with) STAT1	0.791
133	MAPK1 (interacts with) CASP3	0.804
134	MAPK1 (interacts with) FOS	0.877
135	MAPK14 (interacts with) CASP9	0.494
136	MAPK14 (interacts with) F2	0.495
137	MAPK14 (interacts with) IL6	0.53
138	MAPK14 (interacts with) STAT1	0.729
139	MAPK14 (interacts with) CTNNB1	0.753
140	MAPK14 (interacts with) CASP3	0.764
141	MAPK14 (interacts with) NFKBIA	0.766
142	MAPK14 (interacts with) FOS	0.779
143	MAPK14 (interacts with) MAPK1	0.879
144	MMP2 (interacts with) IL10	0.475
145	MMP2 (interacts with) MAPK1	0.437
146	MMP2 (interacts with) TGFBI	0.496
147	MMP2 (interacts with) CASP3	0.568
148	MMP2 (interacts with) IL6	0.627
149	MMP2 (interacts with) CTNNB1	0.633
150	MMP2 (interacts with) HIF1A	0.658
151	MMP9 (interacts with) IL10	0.743
152	MMP9 (interacts with) CASP9	0.553
153	MMP9 (interacts with) MAPK1	0.561
154	MMP9 (interacts with) STAT1	0.474
155	MMP9 (interacts with) FOS	0.574
156	MMP9 (interacts with) IFNG	0.63
157	MMP9 (interacts with) NFKBIA	0.634
158	MMP9 (interacts with) HIF1A	0.655
159	MMP9 (interacts with) F2	0.662
160	MMP9 (interacts with) CASP3	0.759
161	MMP9 (interacts with) CTNNB1	0.791
162	MMP9 (interacts with) IL6	0.851
163	NFKBIA (interacts with) MAPK14	0.766
164	NFKBIA (interacts with) IL10	0.669
165	NFKBIA (interacts with) CASP9	0.566
166	NFKBIA (interacts with) IRF1	0.545
167	NFKBIA (interacts with) MAPK1	0.778
168	NFKBIA (interacts with) BIRC7	0.482
169	NFKBIA (interacts with) MMP9	0.634
170	NFKBIA (interacts with) STAT1	0.681
171	NFKBIA (interacts with) IFNG	0.593
172	NFKBIA (interacts with) IL6	0.85
173	NFKBIA (interacts with) HIF1A	0.473
174	NFKBIA (interacts with) CTNNB1	0.495

175	NFKBIA (interacts with) FOS	0.631
176	NFKBIA (interacts with) CASP3	0.742
177	STAT1 (interacts with) MAPK14	0.729
178	STAT1 (interacts with) IL10	0.823
179	STAT1 (interacts with) IRF1	0.989
180	STAT1 (interacts with) MAPK1	0.791
181	STAT1 (interacts with) MMP9	0.474
182	STAT1 (interacts with) CTNNB1	0.442
183	STAT1 (interacts with) HIF1A	0.447
184	STAT1 (interacts with) IL2	0.564
185	STAT1 (interacts with) CASP3	0.594
186	STAT1 (interacts with) NFKBIA	0.681
187	STAT1 (interacts with) FOS	0.863
188	STAT1 (interacts with) IL6	0.941
189	STAT1 (interacts with) IFNG	0.943
190	TGFBI (interacts with) MMP2	0.496

6 **Table S3 Combine score information of protein-protein interaction (PPI) network**

Table S4

Group	GO term	Gene ratio	pvalue	Count
Biological process	positive regulation of transcription from RNA polymerase II promoter	38.46154	1.90E-08	10
Cellular component	extracellular space	26.92308	5.43E-04	7
Cellular component	cytosol	26.92308	0.001571	7
Molecular function	cytokine activity	15.38462	2.54E-04	4
Molecular function	serine-type endopeptidase activity	15.38462	4.06E-04	4

Table S4 GO terms information

Table S5

GO term	Gene ratio	PValue	Count
Influenza A	30.76923	1.55E-07	8
Toll-like receptor signaling pathway	26.92308	2.86E-07	7
Herpes simplex infection	30.76923	3.02E-07	8
Salmonella infection	23.07692	2.73E-06	6
NOD-like receptor signaling pathway	19.23077	1.06E-05	5
Apoptosis	19.23077	3.28E-05	5
Jak-STAT signaling pathway	19.23077	6.75E-04	5
MAPK signaling pathway	23.07692	7.41E-04	6
Intestinal immune network for IgA production	11.53846	0.005608	3
FoxO signaling pathway	15.38462	0.008963	4
Cytosolic DNA-sensing pathway	11.53846	0.009226	3
Cytokine-cytokine receptor interaction	15.38462	0.015288	4
RIG-I-like receptor signaling pathway	11.53846	0.017007	3
VEGF signaling pathway	11.53846	0.0182	3
p53 signaling pathway	11.53846	0.02069	3

Table S5 KEGG terms information
